# Diversity and Abundance of Ice Nucleating Strains of *Pseudomonas syringae* in a Freshwater Lake in Virginia, USA

**DOI:** 10.3389/fmicb.2017.00318

**Published:** 2017-03-09

**Authors:** Renée B. Pietsch, Boris A. Vinatzer, David G. Schmale

**Affiliations:** ^1^Department of Biological Sciences, Virginia TechBlacksburg, VA, USA; ^2^Department of Plant Pathology, Physiology, and Weed Science, Virginia TechBlacksburg, VA, USA

**Keywords:** ice nucleation, *Pseudomonas*, *Pseudomonas syringae*, *cts*, freshwater bacteria

## Abstract

The bacterium *Pseudomonas syringae* is found in a variety of terrestrial and aquatic environments. Some strains of *P. syringae* express an ice nucleation protein (hereafter referred to as Ice+) allowing them to catalyze the heterogeneous freezing of water. Though *P. syringae* has been sampled intensively from freshwater sources in France, little is known about the genetic diversity of *P. syringae* in natural aquatic habitats in North America. We collected samples of freshwater from three different depths in Claytor Lake, Virginia, USA between November 2015 and June 2016. Samples were plated on non-selective medium (TSA) and on medium selective for *Pseudomonas* (KBC) and closely related species to estimate the total number of culturable bacteria and of *Pseudomonas*, respectively. A droplet freezing assay was used to screen colonies for the Ice+ phenotype. Ice+ colonies were then molecularly identified based on the *cts* (citrate synthase) gene and the 16S rDNA gene. Phylogenetic analysis of *cts* sequences showed a surprising diversity of phylogenetic subgroups of *P. syringae*. Frequencies of Ice+ isolates on *P. syringae* selective medium ranged from 0 to 15% per sample with the highest frequency being found in spring. Our work shows that freshwater lakes can be a significant reservoir of Ice+ *P. syringae*. Future work is needed to determine the contribution of *P. syringae* from freshwater lakes to the *P. syringae* populations present in the atmosphere and on plants and, in particular, if freshwater lakes could be an inoculum source of *P. syringae*-caused plant disease outbreaks.

## Introduction

*Pseudomonas syringae* has been studied as a plant pathogen since the early Twentieth century (Elliott, 1951). It is ubiquitous in most terrestrial environments, including agricultural and uncultivated environments, and aquatic environments (Morris et al., 2013). In regard to aquatic environments, the species has been found in rain, snow, clouds, groundwater, streams, and lakes (Morris et al., 2007, 2008; Vaïtilingom et al., 2012; Renard et al., 2016). While most *P. syringae* strains have the genetic potential to be plant pathogens independently of the environment from which they were isolated, only a minority of strains are known crop pathogens (Monteil et al., 2013).

Some *P. syringae* strains are ice nucleation active (INA) allowing the bacterium to catalyze the freezing of water at temperatures much warmer than the temperature at which pure water freezes. This activity is due to expression of a lipoglycoprotein, called the INA protein (Cochet and Widehem, 2000). Besides *P. syringae*, a variety of inorganic materials, such as dust and minerals, as well as other bacteria, fungi, and pollen can act as ice nuclei (Murray et al., 2012). Ice nuclei including ice-nucleation active bacteria (hereafter referred to as Ice+), such as *P. syringae*, may even contribute to the formation of precipitation in clouds via initiating the crystal lattice structure of ice (Morris et al., 2011; Moukahel et al., 2015). However, not every strain of *P. syringae* has the gene for the INA protein (Lindow, 1983) and natural environmental conditions that favor the expression of the protein in strains with the gene are poorly understood (Nemecek-Marshall et al., 1993). Cold temperatures and low nutrient media tend to favor expression of the protein, but not all strains are induced in the same manner (Nemecek-Marshall et al., 1993).

Recently, a new classification was proposed for *P. syringae* with at least 13 different phylogroups (Berge et al., 2014). Though some strains have been mainly isolated from certain habitats and not others (for example, from aquatic environments but not from crops), many strains have been found in all environments known to be occupied by *P. syringae*. Even strains that are almost identical at the whole genome level have been isolated from crops as well as from aquatic environments suggesting that at least some *P. syringae* lineages are frequently exchanged between aquatic environments and agricultural environments (Monteil et al., 2016).

How the bacteria move with and through the water cycle is not well understood as well as the active and passive roles the bacteria play in this movement. There is evidence that Ice+ bacteria are preferentially found in certain components of the water cycle, such as snow (Morris et al., 2008; Monteil et al., 2012; Joly et al., 2013), and a laboratory experiment showed Ice+ bacteria may aerosolize at higher efficiency from aquatic environments than Ice- bacteria (Pietsch et al., 2015). These findings may be an indication that Ice+ strains of *P. syringae* may be playing an active role in the water cycle. Moreover, Ice+ *P. syringae* may be benefitting from the water cycle as well since the precipitation they induce, may carry bacteria back to nutrient rich environments on the earth surface. This cycle has been described as “bioprecipitation cycle” (Sands et al., 1982; Morris et al., 2014).

Little is known about the relative abundance of *P. syringae* in aquatic environments (Morris et al., 2008). Though studies have been done on distributions of microorganisms in lakes, these have focused on bacterial byproducts in the context of water quality or changes in bacterial communities in response to changes in lake nutrients (McDonough et al., 1986). Lake sediments have also been analyzed for vertical distributions of bacteria (Haglund et al., 2003; Ye et al., 2009). Several studies examined the vertical distribution of bacteria classifying them into broad taxonomic groups (Glöckner et al., 2000; Comeau et al., 2012). Comeau et al. (2012) found γ-Proteobacteria accounted for approximately <2% of the bacteria collected in a lake. The analysis did not classify γ-Proteobacteria further to determine if any *Pseudomonas* were present. Concentrations of bacteria were not determined, but the relative proportion of different types of bacteria changed with depth from 2 to 60 m as well as differing by sampling season (Comeau et al., 2012). To our knowledge, concentration gradients of Ice+ *Pseudomonas syringae* across the water column have not been examined.

The overall goal of this study was to examine the relative abundance of Ice+ strains of *P. syringae* in a large freshwater lake in Virginia, USA. The specific objectives of this study were to: (1) examine the frequency of *P. syringae* within the lake, (2) determine the relative proportion of Ice+ colonies of *Pseudomonas* in the lake, and (3) examine the phylogenetic relationship within and among strains of *P. syringae* collected from the lake. This research has applications in understanding how *P. syringae* is distributed in natural bodies of water, and may work toward greater understanding of the potential for a lake to release aerosolized Ice+ bacteria into the atmosphere.

## Methods and materials

### Sample collection

Samples were collected from Claytor Lake, VA, USA on nine different calendar dates. Fall/winter sampling days were: 5 November 2015, 18 November 2015, 16 December 2015, 21 January 2016, and 4 February 2016. Spring sampling days were: 2 May 2016, 16 May 2016, 25 May 2016, and 7 June 2016. Van Dorn (3.2 L) (Wildco, Yulee, Florida, USA) water samplers were used to collect samples of lake water off the side of a kayak. For each sample, the time, GPS coordinates, and water temperature were recorded (**Table 2**). The samples were taken from four different locations selected to provide a variety of location types and within a reasonable rowing distance from the adjacent beach where the kayak was launched; (1) deep water in the middle of the lake, (2) shallow water near the mouth of an inlet, (3) shallow water in the inlet, (4) the mouth of a boat launch inlet. Samples were collected at the surface of the water at locations 2–4. At location 1, samples were collected at the water surface, at 4.6 and at 9.1 m. Two Van Dorn samplers were used to collect the surface and 4.6 m samples simultaneously in location 1. Table 1 shows the number of samples taken at each location on each sampling day.

**Table 1:** **Five sampling dates with the number of 3.2 Liter water samples collected from Claytor Lake, VA with a Van Dorn sampler at each location**.

**Date**	**Location 1 surface**	**Location 1 4.2 m**	**Location 1 9.1 m**	**Location 2**	**Location 3**	**Location 4**	**Total**
5-Nov-15	1	0	0	1	1	1	4
18-Nov-15	3	0	0	3	0	1	7
16-Dec-15	3	0	0	3	0	0	6
21-Jan-16	3	3	1	0	0	0	7
4-Feb-16	3	3	1	0	0	0	7
2-May-16	3	3	1	0	0	0	7
16-May-16	3	3	3	0	0	0	9
25-May-16	3	3	3	0	0	0	9
2-Jun-16	3	3	3	0	0	0	9
Total	25	18	12	7	1	2	65

*All locations were collected at the water surface, except location 1 where samples were collected at the surface, 4.6 m depth, and 9.1 m depth. Each sample was collected with a 3.2 Liter Van Dorn sampler. See Table 1 for GPS coordinates*.

### Processing of samples for culturable bacteria

The lake water samples were placed on ice in a cooler immediately following collection, and transported back to the laboratory for analysis. In the laboratory, 1 L of each sample was filtered on to 0.2 μm porosity nitrocellulose filters. The filters were placed in 5 mL of the filtrate for 10 min with a stir bar to resuspend the bacteria at a 200X concentration. King's medium B modified with cephalexin (80 mg/L), cycloheximide (200 mg/L), and boric acid (1,500 mg/L) (KBC) (Mohan and Schaad, 1987), selective for *Pseudomonas*, was used to plate 200 μL of the suspension, with three replicates per suspension. The filtrate was also used to make a 2X concentration, which was plated onto 10% tryptic soy agar (TSA) to obtain counts of culturable bacteria which grow on TSA. We define culturable as any bacteria that grew on a given media under environmental conditions of ~20°C. The plates were incubated for 48–72 h at ambient room temperature (~20°C) and the colonies were counted.

### Ice nucleation assays

Colonies that grew on the KBC plates were selected at random (at least 12 colonies per plate) and transferred to 140 μL of water with a sterile toothpick to perform a droplet freezing assay. The samples were stored at 4°C for 24 h. Two droplets of 12 μL of each sample were loaded onto Parafilm® M floating on an Alpha 12 cooling bath (Lauda, New Jersey, USA). Sterile 0.2 μm filtered water was used as a negative control. The temperature of the bath was set at −5°C during loading of the samples, and was then lowered to −12° in 1°C increments. The temperature at which each of the droplets froze was recorded. Strains for which both droplets froze were selected for further analysis.

### Sequence-assisted identification of bacteria

DNA was extracted from cultures using a Puregene Yeast/Bacteria Kit B (Qiagen #1042607) and a BioSprint15 DNA Plant Kit (Qiagen #941517) following manufacturer's protocols. PCR was conducted using a Mastercycler ep Gradient S thermocycler (Eppendorf, New York, USA) with the extracted DNA using primers (forward: 5′ CCC GTC GAG CTG CCA ATW CTG A 3′, reverse: 5′ ATC TCG CAC GGS GTR TTG AAC ATC 3′) for the citrate synthase (*cts*) housekeeping gene to identify colonies of *P. syringae* and related species. A 1% TBE Ethidium Bromide gel was used to visualize products from the PCR reaction. Samples that did not produce a product specific for *P. syringae* were subjected to PCR with 16S primers (518 forward: 5′ CCA GCA GCC GCG GTA ATA CG 3′, 1,491 reverse: 5′ ATC GGY TAC CTT GTT ACG ACT TC 3′). PCR was performed with GoTaq® Green Master Mix (Promega M712) under the following parameters: 1x cycle denaturation 95°C for 10 min, 5x cycle denaturation 95 for 1 min, annealing 53°C for 30 s, extension 72°C 1 min 40 s, 25x cycle 94°C 30 s, 53°C 20 s, 72°C 1 min, 1x cycle 72°C 10 min, hold 4°C). The samples were purified using ExoI/rSAP prior to sequencing (AB1 3730x1 DNA Sequencer, Eton Biosciences, 104 T.W. Alexander Drive, Bldg 4A, RTP, NC 27709, USA). The sequences from 16S primers were used to search GenBank (http://www.ncbi.nlm.nih.gov/blast) using the Blastn algorithm to identify the sequence with the closest match. The sequences from *cts* primers along with 38 reference strains of *P. syringae* (Berge et al., 2014) were aligned using the Clustal W method and a phylogenetic tree was constructed selecting the neighbor-joining algorithm in MEGA7 with 1,000 bootstrap replicates.

### Statistical analyses

Statistical analyses were conducted in R (Version 3.3.1, http://www.r-project.org). Because data were not normally distributed, non-parametric Kruskal-Wallis rank sum tests, were used to test for significant differences (*P* < 0.05) between percent of Ice+ strains between colony forming units (CFU) of samples at different depths (*n* = 55) and locations (*n* = 17). A Wilcoxon rank sum test for non-parametric data was used to compare between percent of Ice+ strains in fall/winter and spring collections (*n* = 66).

## Results

### Concentration of bacteria

**Figure 1:**
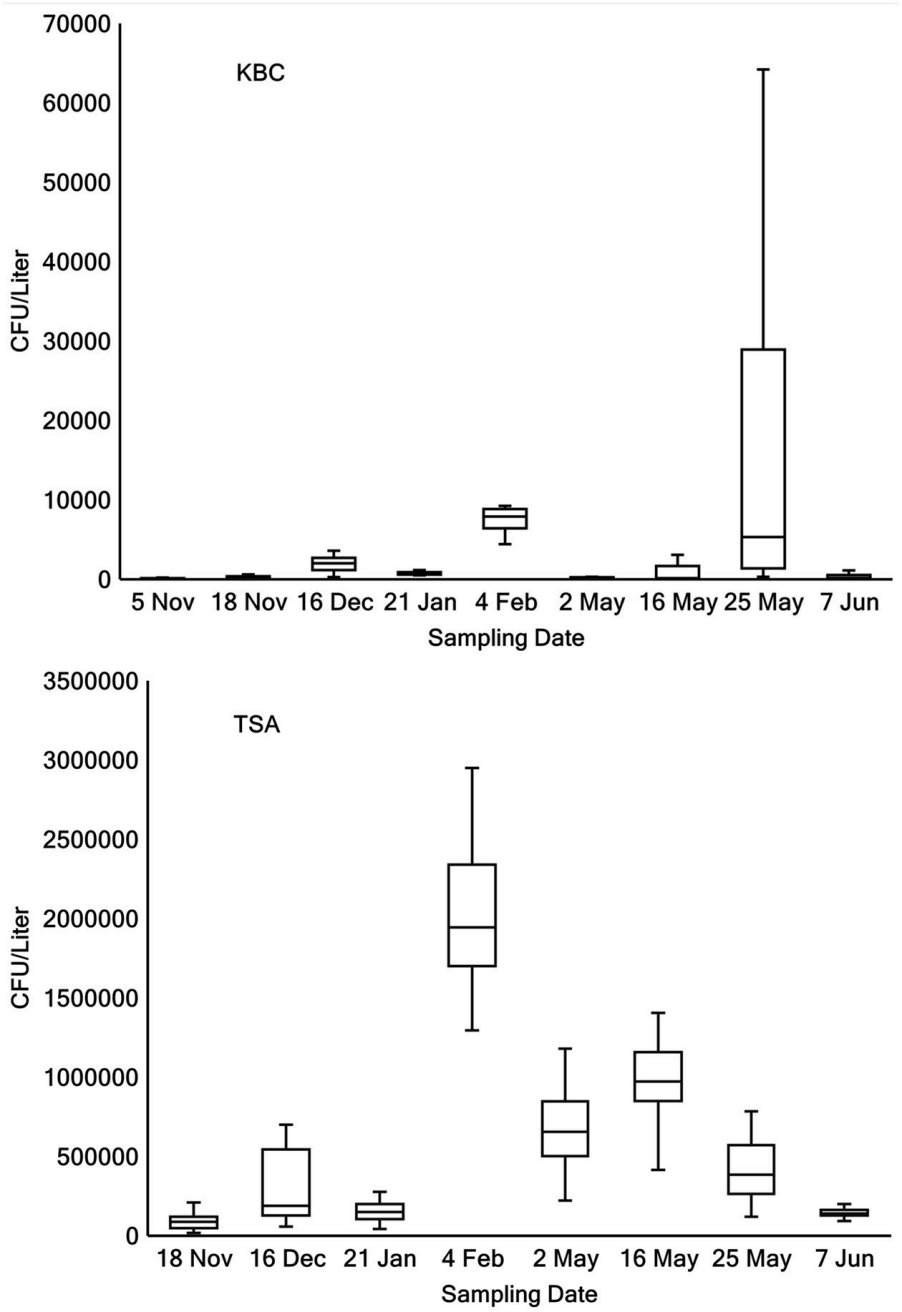
**Box plots of concentrations (CFU Liter^−1^) of *Pseudomonas* grown on KBC (top panel)** and bacteria grown on TSA **(bottom panel)** across all locations on each day of sampling.

The concentration of bacteria (CFU Liter^−1^) and *Pseudomonas* in lake water was estimated from the counts of colonies on TSA and KBC media, respectively (Table 2). Between 0 and 30% of the bacteria cultured on TSA were *Pseudomonas*. In 17 of 66 samples, between 1 and 10% of the strains tested were Ice+, and in 16 of 66 samples >10% of the strains tested were Ice+. The concentration of colonies that grew on KBC media (*Pseudomonas)* for each sampling location for each day of collection showed considerable variation across all days and across all sampling locations. Figure 1 shows box plots of the concentration (CFU Liter^−1^) of *Pseudomonas* from all locations that grew on KBC media for all days of sampling, indicating that *Pseudomonas* was consistently found throughout Claytor Lake. However, the mean concentration for each collection day varied dramatically from a low of 110 CFU Liter^−1^ on 5 November 2015 to 16,240 CFU Liter^−1^ on 25 May 2016.

**Table 2:** **Data for each 3.2 Liter water sample collected from Claytor Lake, VA with a Van Dorn sampler including sampling date, ambient air temperature (°F), water temperature at sampling location (°F), sampling depth, GPS location, Mean CFU Liter^−1^ of *Pseudomonas* grown on KBC media based on three plates, mean CFU Liter^−1^ of total bacteria on TSA media based on three plates, percent *Pseudomonas* grown on KBC media, percent of Ice+ strains from colonies grown on KBC media**.

**Sampling date**	**Air temp (°C)**	**Water temp (°C)**	**Location area**	**Sample depth**	**GPS coordinates**	**Mean CFU liter^−1^ on KBC media ± st. dev**.	**Mean CFU Liter^−1^ on TSA media ± st. dev**.	**Ratio of CFU on KBC:TSA (%)**	**%Ice+ colonies**
5-Nov-15	18.9	n.d.	4	Surface	37.054566, −80.620337	192 ± 88	n.d.	n.d.	10.34
5-Nov-15	18.9	n.d.	2	Surface	37.051167, −80.623722	100 ± 25	n.d.	n.d.	43.75
5-Nov-15	18.9	n.d.	3	Surface	37.05208, −80.624204	108 ± 104	n.d.	n.d.	26.32
5-Nov-15	18.9	n.d.	1	Surface	37.052242, −80.617239	42 ± 29	n.d.	n.d.	0.00
5-Nov-15	18.9	n.d.	1	3.0 m	37.052242, −80.619078	25 ± 0	n.d.	n.d.	4.17
18-Nov-15	11.1	n.d.	2	Surface	37.050904, −80.623690	367 ± 138	9.42E+04 ± 629	0.39	0.00
18-Nov-15	11.1	n.d.	2	Surface	37.051063, −80.623709	208 ± 166	8.50E+04 ± 444	0.25	5.56
18-Nov-15	11.1	n.d.	2	Surface	37.051246, −80.623684	50 ± 43	2.75E+04 ± 152	0.18	0.00
18-Nov-15	11.1	n.d.	1	Surface	37.051911, −80.619642	283 ± 159	8.42E+04 ± 603	0.34	5.88
18-Nov-15	11.1	n.d.	1	Surface	37.052095, −80.619428	442 ± 101	7.92E+04 ± 506	0.56	20.69
18-Nov-15	11.1	n.d.	1	Surface	37.052487, −80.619154	525 ± 361	1.25E+05 ± 777	0.42	32.00
18-Nov-15	11.1	n.d.	4	Surface	37.054582, −80.620274	267 ± 101	1.70E+05 ± 955	0.16	7.69
16-Dec-15	8.9	9.4	2	Surface	37.051218, −80.623385	2, 133 ± 188	3.03E+05 ± 961	0.71	7.35
16-Dec-15	8.9	9.5	2	Surface	37.050978, −80.623684	2, 767 ± 540	5.93E+05 ± 595	0.47	17.86
16-Dec-15	8.9	9.4	2	Surface	37.051368, −80.623841	3, 500 ± 87	6.20E+05 ± 826	0.57	10.29
16-Dec-15	8.9	9.1	1	Surface	37.052293, −80.619069	1, 700 ± 400	1.11E+05 ± 311	1.53	14.71
16-Dec-15	8.9	9.0	1	Surface	37.052179, −80.619059	1, 167 ± 161	1.68E+05 ± 247	0.69	16.67
16-Dec-15	8.9	9.4	1	Surface	37.052249, −80.619043	400 ± 115	7.08E+04 ± 138	0.57	13.46
21-Jan-16	−1.1	40.7	1	4.2 m	37.0515635, −80.6169486	717 ± 188	1.00E+05 ± 50	0.72	10.87
21-Jan-16	−1.1	4.6	1	Surface	37.0509283, −80.6169794	583 ± 76	9.33E+04 ± 454	0.63	4.84
21-Jan-16	−1.1	4.6	1	4.2 m	37.0522448, −80.6170897	967 ± 151	2.19E+05 ± 601	0.44	10.29
21-Jan-16	−1.1	4.6	1	Surface	37.0522448, −80.6170897	683 ± 104	1.41E+05 ± 322	0.49	8.33
21-Jan-16	−1.1	4.2	1	4.2 m	37.0520847, −80.6169546	700 ± 150	2.14E+05 ± 454	0.33	12.50
21-Jan-16	−1.1	4.6	1	Surface	37.0517267, −80.6169077	692 ± 161	1.10E+05 ± 402	0.63	9.62
21-Jan-16	−1.1	4.5	1	9.1 m	37.052242, −80.617239	1, 083 ± 63	1.96E+05 ± 118	0.55	11.76
4-Feb-16	5.6	2.0	1	4.2 m	37.0522073, −80.6180414	7, 750 ± 238	1.74E+06 ± 2,480	0.45	1.79
4-Feb-16	5.6	2.1	1	Surface	37.0520805, −80.6184315	4, 942 ± 480	1.51E+06 ± 2,040	0.33	7.69
4-Feb-16	5.6	1.9	1	4.2 m	37.0520144, −80.6188472	7, 800 ± 327	1.72E+06 ± 1400	0.45	13.33
4-Feb-16	5.6	2.1	1	Surface	37.0519812, −80.6188476	5, 875 ± 517	2.30E+06 ± 779	0.26	8.33
4-Feb-16	5.6	2.0	1	4.2 m	37.0521998, −80.6190779	8, 792 ± 347	2.36E+06 ± 2,880	0.37	3.85
4-Feb-16	5.6	2.1	1	Surface	37.0521254, −80.6189773	9, 000 ± 312	1.89E+06 ± 3,760	0.48	12.50
4-Feb-16	5.6	2.0	1	9.1 m	37.0522846, −80.6189418	9, 092 ± 210	2.79E+06 ± 1,760	0.33	0.00
2-May-16	22.8	20.9	1	Surface	n.d.	183 ± 80	1,015,833 ± 1,420	0.02	0.00
2-May-16	22.8	20.3	1	Surface	n.d.	67 ± 29	487,500 ± 180	0.01	0.00
2-May-16	22.8	19.6	1	4.2 m	n.d.	217 ± 101	647,500 ± 66	0.03	8.00
2-May-16	22.8	20.6	1	Surface	n.d.	175 ± 66	904,167 ± 521	0.02	0.00
2-May-16	22.8	19.5	1	4.2 m	n.d.	542 ± 38	675,000 ± 541	0.08	0.00
2-May-16	22.8	19.9	1	4.2 m	n.d.	208 ± 38	655,833 ± 1,250	0.03	4.16
2-May-16	22.8	n.d.	1	9.1 m	n.d.	83 ± 38	270,000 ± 426	0.03	0.00
16-May-16	10	17.7	1	Surface	n.d.	42, 200 ± 5, 495	410,000 ± 303	10.29	0.00
16-May-16	10	18.9	1	4.2 m	n.d.	92 ± 63	1,227,500 ± 180	0.01	9.09
16-May-16	10	18.9	1	Surface	n.d.	1, 225 ± 229	898,333 ± 440	0.14	0.00
16-May-16	10	18.9	1	4.2 m	n.d.	183 ± 52	889,167 ± 1,620	0.02	4.34
16-May-16	10	18.9	1	9.1 m	37.0522500, −80.6190833	8 ± 14	1,052,500 ± 2,870	0.001	0.00
16-May-16	10	18.9	1	9.1 m	37.0522500, −80.6190833	2, 908 ± 153	1,018,333 ± 3,570	0.29	0.00
16-May-16	10	18.9	1	Surface	37.0522500, −80.6190278	2, 433 ± 95	1,140,833 ± 2,040	0.21	0.00
16-May-16	10	18.9	1	4.2 m	37.0522500, −80.6190278	42 ± 14	960,833 ± 1,030	0.004	0.00
16-May-16	10	18.9	1	9.1 m	37.0522222, −80.6190556	83 ± 76	995,833 ± 571	0.01	0.00
25-May-16	18.9	19.7	1	Surface	n.d.	57, 000 ± 7, 632	188,333 ± 1,180	30.27	0.00
25-May-16	18.9	18.8	1	4.2 m	n.d.	6, 775 ± 1, 233	415,000 ± 557	1.63	0.00
25-May-16	18.9	19.9	1	Surface	n.d.	1, 358 ± 232	385,833 ± 313	0.35	0.00
25-May-16	18.9	18.8	1	4.2 m	n.d.	2, 033 ± 281	642,500 ± 1,230	0.32	0.00
25-May-16	18.9	*n*.*d*.	1	Surface	n.d.	6, 025 ± 876	407,500 ± 1,100	1.48	0.00
25-May-16	18.9	*n*.*d*.	1	4.2 m	n.d.	500 ± 152	700,833 ± 525	0.07	0.00
25-May-16	18.9	*n*.*d*.	1	9.1 m	n.d.	1, 142 ± 213	772,500 ± 3,830	0.15	0.00
25-May-16	18.9	*n*.*d*.	1	9.1 m	n.d.	39, 917 ± 3, 894	192,500 ± 90	20.74	0.00
25-May-16	18.9	*n*.*d*.	1	9.1 m	n.d.	31, 408 ± 4, 367	246,667 ± 32	12.73	0.00
7-Jun-16	21.1	24.9	1	Surface	37.0524355, −80.616631	38, 167, 364	144,167 ± 184	26.47	0.00
7-Jun-16	21.1	24.9	1	4.2 m	37.0524355, −80.616631	0 ± 0	155,833 ± 218	0.00	0.00
7-Jun-16	21.1	21.7	1	9.1 m	n.d.	383 ± 38	250,833 ± 38	0.15	2.20
7-Jun-16	21.1	21.7	1	9.1 m	n.d.	10, 500 ± 90	155,833 ± 392	6.74	0.00
7-Jun-16	21.1	25.0	1	Surface	37.0525921667, −80.617109333	4, 167 ± 216	119,167 ± 191	3.50	0.00
7-Jun-16	21.1	24.9	1	4.2 m	37.0525921667, −80.617109333	42 ± 52	113,333 ± 201	0.04	0.00
7-Jun-16	21.1	24.8	1	9.1 m	n.d.	17 ± 29	192,500 ± 1, 040	0.01	0.00
7-Jun-16	21.1	25.2	1	Surface	37.0523113333, −80.6172223333	142 ± 14	139,167 ± 227	0.10	0.00
7-Jun-16	21.1	25.0	1	4.2 m	37.0523113333, −80.6172223333	8 ± 14	140,000 ± 152	0.01	0.00

*n.d. = no data*.

### Ice+ *Pseudomonas* strains

**Figure 2:**
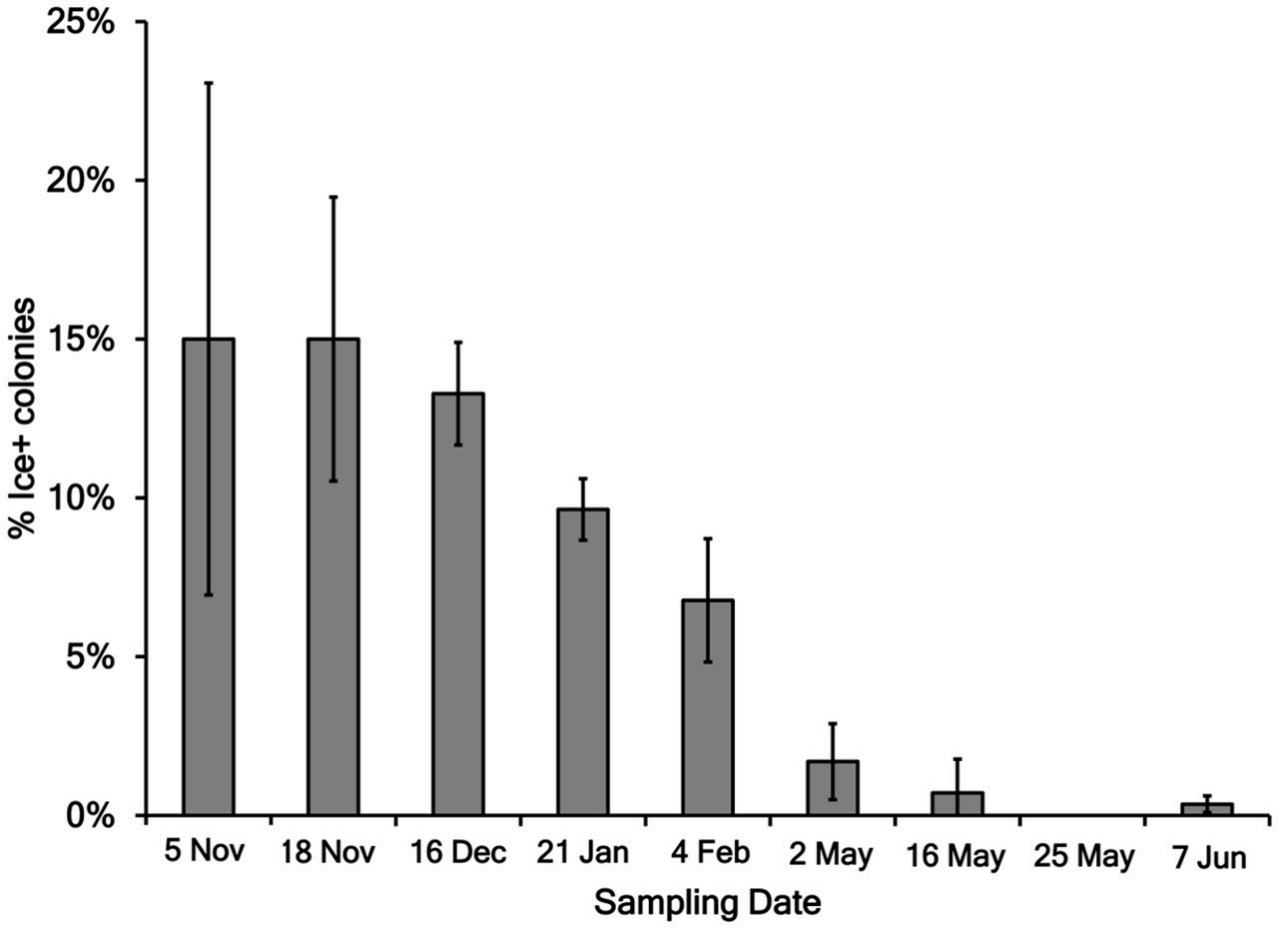
**Percent of Ice+ *Pseudomonas* colonies grown on KBC media for each day of sampling**. Error bars are standard error of the water samples on each collection day. The following number of droplets were tested for each collection day: 5 November 2015 = 160, 18 November 2015 = 220, 16 December 2015 = 384, 21 January 2016 = 384, 4 February 2016 = 384, 2 May 2016 = 177, 16 May 2016 = 283, 25 May 2016 = 476, and 7 June 2016 = 282.

The 2,750 colonies from KBC media were tested for the Ice+ phenotype with an ice nucleation assay. The freezing temperature between −5°C and −12°C was recorded for each droplet, and 58% froze at −5°C, 14% at −6°C, 4% at −7°C, 3% at −8°C, 4% at −9°C, 7% at −10°C, 5% at −11°C, and 6% at −12°C. The percent of colonies for which both tested droplets froze (Table 3, Figure 2) was between 0 and 15% of the colonies on all sampling days. A total of 176 strains was Ice+ (Table 4). A majority of the Ice+ samples produced a product with the *cts* primer pair indicating they were *P. syringae* (Table 4). The percentage of Ice+ *Pseudomonas* colonies that did not produce a *cts* PCR product from the fall/winter collections (therefore, likely not *P. syringae*) was between 15 and 20% on each day of sampling with the exception of 4 February 2016 for which 47% of the Ice+ colonies were not *P. syringae*. The spring collections were not considered because the sample size of Ice+ strains was too small to obtain an accurate percentage. The 16S sequence results confirmed that these colonies were not *P. syringae;* they were identified as *Xanthomonas, P. fragi, P. fluorescens, P. viridiflava*, and *P. plecoglossicida*.

**Table 3:** **The number of *Pseudomonas* colonies from the KBC media that froze for each day of sampling using an ice nucleation assay**.

**Sampling date**	**Number of Ice+ colonies**	**Number of colonies assayed**	**% of colonies Ice+**
5 November 2015	24	160	15.0
18 November 2015	33	220	15.0
16 December 2015	51	384	13.3
21 January 2016	37	384	9.6
4 February 2016	26	384	6.8
2 May 2016	3	177	1.7
16 May 2016	2	283	0.7
25 May 2016	0	476	0.0
7 June 2016	1	282	0.4

**Table 4:** **Freezing temperatures of replicates 1 and 2 for each strain tested for ice nucleation activity, and GenBank accession numbers for cts sequences for strains of *P. syringae***.

**Strain ID**	**Freezing temp of droplet #1 (°C)**	**Freezing temp of droplet #2 (°C)**	**Sequence ID**	**Gen Bank #**
CLA 1	−8	−8	*P. syringae*	KY629082
CLA 2	−5	−5	*P. syringae*	KY629083
CLA 3	−5	−5	*P. syringae*	KY629084
CLA 4	−5	−5	*P. syringae*	KY629085
CLA 5	−5	−5	*P. syringae*	KY629086
CLA 6	−5	−5	*P. syringae*	KY629087
CLA 7	−5	−5	*P. syringae*	KY629088
CLA 8	−5	−5	*P. syringae*	KY629089
CLA 9	−6	−5	*P. fragi*	−
CLA 10	−5	−5	*P. syringae*	KY629090
CLA 11	−5	−5	*P. syringae*	KY629091
CLA 12	−5	−5	*P. syringae*	KY629092
CLA 13	−11	−10	*P. syringae*	KY629093
CLA 14	−5	−5	*P. syringae*	KY629094
CLA 15	−5	−5	*P. syringae*	KY629095
CLA 16	−5	−5	*P. syringae*	KY629096
CLA 17	−7	−7	*P. syringae*	KY629097
CLA 18	−10	−9	*P. syringae*	KY629098
CLA 19	−5	−5	*P. syringae*	KY629099
CLA 20	−7	−9	*Xanthomonas*	−
CLA 21	−5	−5	*P. syringae*	KY629100
CLA 22	−10	−11	*P. syringae*	KY629101/102
CLA 23	−11	−11	*Xanthomonas*	−
CLA 24	−11	−12	*Xanthomonas*	−
CLB1	−5	−5	*P. syringae*	KY629103
CLB2	−5	−5	*P. syringae*	KY629104
CLB3	−6	−6	*P. syringae*	KY629105
CLB4	−5	−5	*P. syringae*	KY629106
CLB5	−6	−7	*P. syringae*	KY629107
CLB6	−5	−6	*P. syringae*	KY629108
CLB7	−10	−10	*P. syringae*	KY629109
CLB8	−5	−5	*P. syringae*	KY629110
CLB9	−5	−5	*P. syringae*	KY629111
CLB10	−8	−11	*P. fluorescens*	−
CLB11	−5	−5	*P. syringae*	KY629112
CLB12	−5	−5	*P. syringae*	KY629113
CLB13	−7	−7	*P. syringae*	KY629114
CLB14	−5	−5	*P. fragi*	−
CLB15	−5	−5	*P. syringae*	KY629115
CLB16	−5	−5	*P. syringae*	KY629116
CLB17	−5	−5	*P. syringae*	KY629117
CLB18	−5	−5	*P. syringae*	KY629118
CLB19	−5	−5	*P. syringae*	KY629119
CLB20	−5	−5	*P. syringae*	KY629120
CLB21	−5	−5	*P. syringae*	KY629121
CLB22	−5	−5	*P. syringae*	KY629122
CLB23	−5	−5	*P. syringae*	KY629123
CLB24	−5	−5	*P. syringae*	KY629124
CLB25	−5	−5	*P. syringae*	−
CLB26	−5	−5	*P. syringae*	KY629125
CLB27	−11	−11	*P. syringae*	KY629126
CLB28	−5	−8	*P. syringae*	KY629127
CLB29	−5	−5	*P. fluorescens*	KY629128
CLB30	−10	−7	*P. syringae*	KY629129
CLB31	−5	−5	*P. fragi*	−
CLB32	−11	−9	*P. fragi*	−
CLC1	−5	−5	*P. syringae*	KY629130
CLC2	−5	−5	*P. syringae*	KY629131
CLC3	−9	−9	*P. syringae*	KY629132
CLC4	−5	−5	*P. syringae*	KY629133
CLC5	−5	−5	*P. syringae*	KY629134
CLC6	−6	−12	*P. fluorescens*	−
CLC7	−5	−5	*P. syringae*	KY629135
CLC8	−5	−6	*P. syringae*	KY629136
CLC9	−5	−7	*P. syringae*	KY629137
CLC10	−6	−5	*P. syringae*	KY629138
CLC11	−11	−10	*P. fragi*	−
CLC12	−7	−5	*P. viridiflava*	KY629139
CLC13	−6	−5	*P. syringae*	KY629140
CLC14	−6	−5	*P. syringae*	KY629141
CLC15	−6	−5	*P. syringae*	KY629142
CLC16	−5	−5	*P. syringae*	KY629143
CLC17	−6	−5	*P. syringae*	KY629144
CLC18	−6	−5	*P. syringae*	KY629145
CLC19	−6	−5	*Pseudomonas*	−
CLC20	−6	−5	*P. syringae*	KY629146
CLC21	−6	−8	*P. syringae*	KY629147
CLC22	−6	−9	*P. syringae*	KY629148
CLC23	−5	−5	*P. syringae*	KY629149
CLC24	−5	−5	*P. viridiflava*	−
CLC25	−5	−5	*P. syringae*	KY629150
CLC26	−5	−5	*P. syringae*	KY629151
CLC27	−5	−5	*P. syringae*	KY629152
CLC28	−12	−7	*P. syringae*	KY629153
CLC29	−5	−6	*P. syringae*	KY629154
CLC30	−5	−6	*P. syringae*	KY629155
CLC31	−10	−14	*P. syringae*	KY629156
CLC32	−12	−13	*Pseudomonas*	−
CLC33	−8	−14	*P. fragi*	KY629157
CLC34	−5	−6	*P. syringae*	KY629158
CLC35	−12	−12	*P. fragi*	KY629159
CLC36	−12	−12	*P. syringae*	KY629160
CLC37	−5	−6	*P. syringae*	KY629161
CLC38	−5	−7	*P. syringae*	KY629162
CLC39	−6	−5	*P. syringae*	KY629163
CLC40	−5	−5	*P. syringae*	KY629164
CLC41	−5	−5	*P. syringae*	KY629165
CLC42	−12	−7	*P. syringae*	KY629166
CLC43	−6	−5	*P. viridiflava*	−
CLC44	−6	−5	*P. syringae*	KY629167
CLC45	−6	−5	*P. syringae*	KY629168
CLC46	−6	−5	*P. syringae*	KY629169
CLC47	−6	−5	*P. syringae*	KY629170
CLC48	−11	−5	*P. syringae*	KY629171
CLC49	−9	−11	*P. syringae*	KY629172
CLC50	−6	−5	*P. syringae*	KY629173
CLC51	−12	−9	*Pseudomonas*	−
CLD1	−5	−5	*P. syringae*	KY629174
CLD2	−5	−5	*P. syringae*	KY629175
CLD3	−5	−5	*P. syringae*	KY629176
CLD4	−5	−5	*P. syringae*	KY629177
CLD5	−5	−5	*P. syringae*	KY629178
CLD6	−5	−5	*P. syringae*	−
CLD7	−5	−5	*P. syringae*	KY629179
CLD8	−5	−5	*P. syringae*	KY629180
CLD9	−12	−12	*P. syringae*	KY629181
CLD10	−6	−6	*P. syringae*	KY629182
CLD11	−6	−6	*P. syringae*	KY629183/184
CLD12	−12	−9	*P. syringae*	−
CLD13	−5	−5	*P. syringae*	KY629185
CLD14	−5	−5	*P. fragi*	−
CLD15	−5	−5	*P. syringae*	KY629186
CLD16	−5	−5	*P. syringae*	KY629187
CLD17	−5	−5	*P. syringae*	KY629188
CLD18	−5	−5	*P. syringae*	KY629189
CLD19	−6	−5	*P. syringae*	KY629190
CLD20	−6	−5	*P. syringae*	KY629191
CLD21	−5	−5	*P. syringae*	KY629192
CLD22	−6	−6	*P. syringae*	KY629193
CLD23	−6	−6	*P. syringae*	KY629194
CLD24	−6	−6	*P. plecoglossicida*	KY629195
CLD25	−6	−6	*P. syringae*	KY629196
CLD26	−6	−6	*P. syringae*	KY629197
CLD27	−5	−5	*P. syringae*	KY629198
CLD28	−5	−5	*P. syringae*	KY629199
CLD29	−11	−11	*P. syringae*	KY629200
CLD30	−5	−5	*P. syringae*	KY629201
CLD31	−5	−5	*Pseudomonas*	KY629202
CLD32	−5	−5	*P. syringae*	−
CLD33	−11	−11	*P. fragi*	−
CLD34	−11	−10	*P. syringae*	KY629203
CLD35	−9	−9	*Pseudomonas*	−
CLD36	−6	−6	*P. syringae*	KY629204
CLD37	−6	−5	*P. syringae*	KY629205/206
CLE1	−12	−11	*P. fragi*	−
CLE2	−8	−10	*P. fragi*	−
CLE3	−5	−5	*P. syringae*	KY629207
CLE4	−10	−10	*P. syringae*	KY629208
CLE5	−8	−10	*P. fragi*	−
CLE6	−6	−6	*P. syringae*	KY629209
CLE7	−5	−5	*Pseudomonas*	−
CLE8	−12	−7	*P. fragi*	−
CLE9	−5	−5	*P. viridiflava*	−
CLE10	−5	−5	*P. fragi*	−
CLE11	−5	−5	*P. syringae*	KY629210
CLE12	−5	−12	*P. syringae*	KY629211
CLE13	−5	−5	*P. syringae*	KY629212
CLE14	−10	−10	*Pseudomonas*	−
CLE15	−10	−10	*Pseudomonas*	−
CLE16	−7	−5	*Pseudomonas*	−
CLE17	−12	−12	*Pseudomonas*	−
CLE18	−10	−10	*P. fragi*	−
CLE19	−5	−5	*P. syringae*	KY629213
CLE20	−8	−10	*P. syringae*	KY629214
CLE21	−5	−5	*P. syringae*	KY629215
CLE22	−5	−5	*P. syringae*	KY629216
CLE23	−10	−10	*P. syringae*	KY629217
CLE24	−5	−5	*P. syringae*	KY629218
CLE25	−5	−5	*P. syringae*	KY629219
CLE26	−5	−5	*P. syringae*	KY629220
CLF1	−5	−5	*P. syringae*	KY629221
CLF2	−5	−5	*P. syringae*	KY629222
CLF3	−9	−9	*P. syringae*	KY629223
CLG1	−5	−5	*P. syringae*	KY629224
CLG2	−5	−5	*Pseudomonas*	−
CLI1	−7	−10	*P. syringae*	KY629225

*CLA, November 2015; CLB, 18 November 2015; CLC, 16 December 2015; CLD, 21 January 2016; CLE, 4 February 2016; CLF, 2 May 2016; CLG, 16 May 2016; CLI, 7 June 2016*.

### Phylogenetic analysis of *Pseudomonas syringae* strains

**Figure 3:**
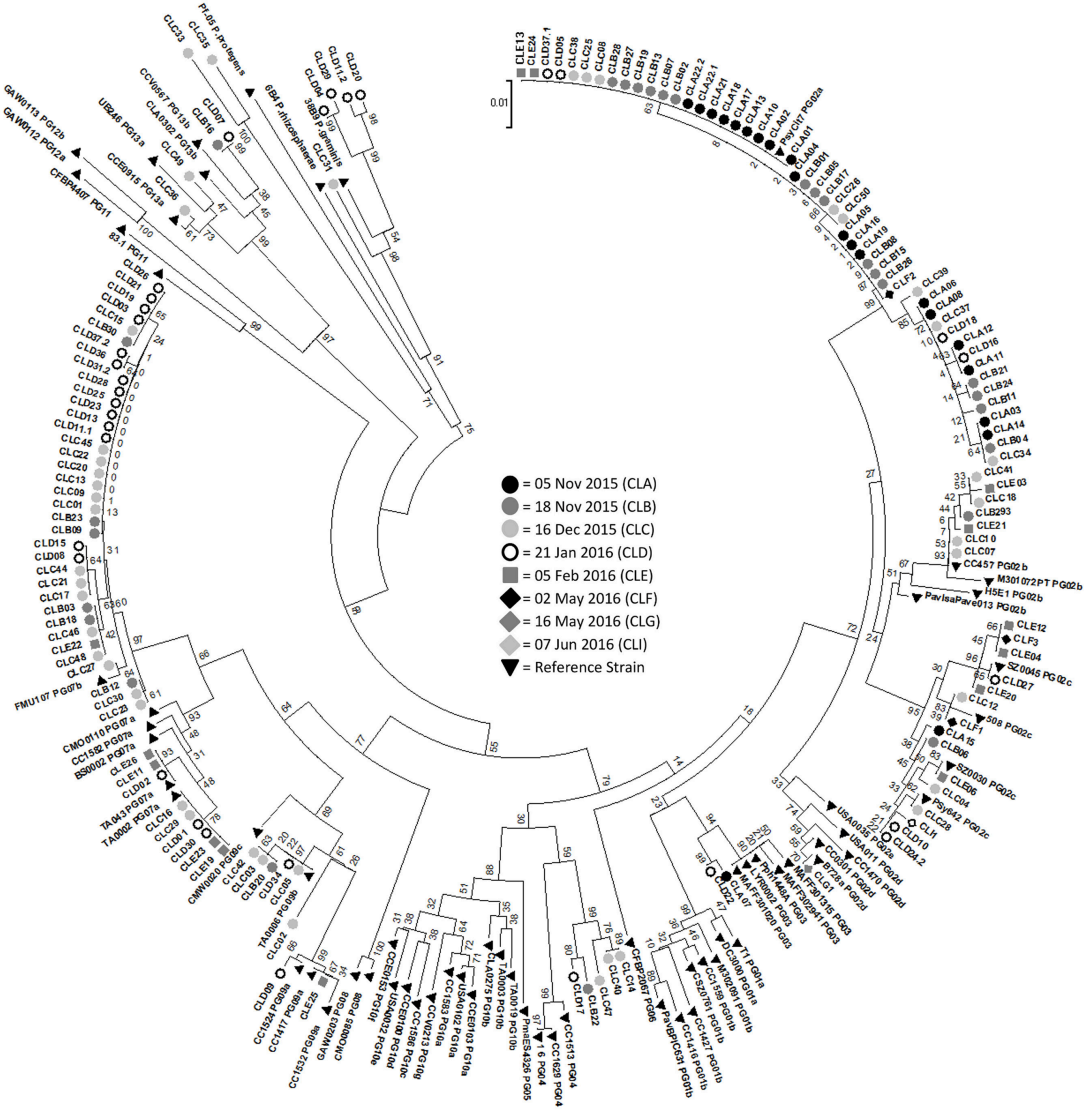
**Phylogenetic tree with samples from all days of sampling along with 38 reference strains representing the diversity of *Pseudomonas syringae* strains**. Bootstrap values are listed. 5 November 2015 = CLA (black circle), 18 November 2015 = CLB (dark gray circle), 16 December 2015 = CLC (light gray circle), 21 January 2016 = CLD (white circle), 4 February 2016 (medium gray square) = CLE, 2 May 2016 (black diamond) = CLF, 16 May 2016 (medium gray diamond) = CLG, 7 June 2016 (white diamond) = CLI. Reference strains = black triangle. The phylogroup of the reference strains is marked by PG followed by the number of the phylogroup.

A phylogenetic tree was constructed with all of the samples that produced a *cts* product as well as 38 reference strains representing 13 phylogroups of *P. syringae* (Berge et al., 2014; Figure 3). The strains from Claytor Lake were compared to the 13 phylogroups with all groupings having bootstrap values of 84 or higher. The samples from Claytor Lake represent six phylogroups groups: 2, 3, 4, 7, 9, and 13. The 5 November 2015 collection showed less diversity, with all isolates belonging to phylogroup 2 and one isolate belonging to phylogroup 3. This is the only sample collection that included samples from location 3 and 4. Thus, all isolates from these two locations are in phylogroup 2 and 3.

### Non-*Pseudomonas syringae* strains

The colonies that did not produce a *cts* product were sequenced with 16S primers and BLAST results showed they were all *Pseudomonas* with the exception of three colonies which represented members of the genus *Xanthomonas*. The BLAST results for the *Pseudomonas* colonies that were not *P. syringae* showed a 100% match with *P. fragi, P. fluorescens, P. viridiflava*, and *P. plecoglossicida*. Additional phylogenetic analyses are needed to confirm these taxonomic assignments, which is beyond the scope of this study.

### Statistical analyses

There was no significant difference between the CFU on KBC media at different depths (*P* = 0.913) or locations (*P* = 0.567). There was a significant difference between the percent of Ice+ strains in the fall collections and spring collections (*P* < 0.001).

## Discussion

Little is known about the abundance of *Pseudomonas* in aquatic environments. Here, we show the distribution of *Pseudomonas* in a freshwater lake, examining the concentration across sampling dates in two seasons and at three depths. *Pseudomonas* colonies were obtained from every sample collected on every day of sampling, indicating they are ubiquitous throughout Claytor Lake. Variation was observed in the concentration of *Pseudomonas* at different sampling locations and across different sampling days. The concentrations of *Pseudomonas* collected ranged widely, between 25 and 5.7 × 10^5^ CFU Liter^−1^ indicating a non-uniform distribution. Within each day of sampling there was variation between sampling locations. The location with the highest concentration varied from day to day, suggesting that bacteria continually move and mix and/or reproduce and die at different rates at different locations depending on local environmental conditions. Comparisons to previous concentrations are limited due to a lack of reported concentrations of *Pseudomonas* in freshwater lakes. However, Morris et al. (2008) sampled four freshwater lakes and found similar concentrations of *Pseudomonas syringae* between 130 and 1 × 10^4^ CFU Liter^−1^.

The relative abundance of *Pseudomonas* ranged between 0 and 30% of the total culturable bacteria (based on counts from TSA), with most collections between 0.1 and 1.5%, indicating *Pseudomonas* was a small minority of the bacteria present. The concentration of *Pseudomonas* appears to be variable and subject to change day to day and even in different locations on the same day. Previous studies examining the relative abundances of bacteria in freshwater lakes found between 5 and 10% of the bacteria sampled were γ-Proteobacteria (which includes *Pseudomonas*; Hiorns et al., 1997; Tamaki et al., 2005; Briée et al., 2007; Wang et al., 2012).

There was no significant difference in the concentration of *Pseudomonas* at different depths (*P* = 0.913) or locations (*P* = 0.567). The first 2 days of sampling five different locations in the lake were sampled, but with this finding of non-significance one location was chosen for the remaining 7 days of sampling. In general, more variation was seen in the concentrations of CFU on KBC media in the spring collections since both, the highest and lowest concentrations, were obtained in the spring collections. The total bacteria count on non-selective TSA medium did not show as much variation between the two seasons of collection, suggesting that *Pseudomonas* is likely more subject to variation than the bacteria cultured on TSA.

Between 0 and 15% of the *Pseudomonas* colonies were Ice+ on each sampling day, indicating Ice+ strains are consistently present throughout Claytor Lake. The Ice+ colonies were not evenly distributed across samples on a given day. Some collections did not have any Ice+ colonies and others had up to 50%. Joly et al. (2013) sampled cloud water for Ice+ *Pseudomonas* and found 12% of the strains tested were Ice+. In regard to ice nucleation activity, the populations of *Pseudomonas* in Claytor Lake and in clouds are similar, though different methods were used to test for ice nucleation activity in these different environments. Strains of *Pseudomonas* may be moving between clouds and freshwater lakes via rain and aerosolization (Morris et al., 2014), but experiments to track specific strains moving within and among these environments have not yet been conducted. There was a significant difference between the percent of Ice+ strains in the fall collections and spring collections (*P* < 0.001). The five fall/winter collections had a higher frequency of Ice+ bacteria ranging from 6 to 15% while the spring collections had a frequency of 0–2%. This significant difference between these collections suggests a potential ecological role for the ice nucleation phenotype; Ice+ strains of *P. syringae* may be better adapted to colder climates.

Strains of Ice+ *P. syringae* were identified from diverse phylogroups. Berge et al. (2014) conducted phylogenetic analysis using 4 housekeeping genes from 763 strains of *P. syringae* and found that using only the *cts* gene sequence was a reliable and efficient method of classifying *P. syringae*. Berge et al. (2014) examined the phenotypic and genetic characteristics of strains of *P. syringae* within each of 13 phylogroups. Many of the strains from Claytor Lake were in phylogroup 2, which is the most widespread phylogroup found in all habitats studied with around 85% of the strains previously sampled being Ice+ (Morris et al., 2010). Two strains were in phylogroup 3, which includes many crop pathogens although few strains from this phylogroup have been found in the environment perhaps due to competition from faster growing strains (Monteil et al., 2014). About 20% of previously sampled phylogroup 3 strains were Ice+ (Berge et al., 2014). Five strains may be in phylogroup 4, although there was weak bootstrap support for this grouping at only 60. All strains previously reported in phylogroup 4 have been Ice+ although these strains are rarely detected in the environment (Berge et al., 2014). Many of the strains from Claytor Lake were in phylogroup 7, which includes strains previously identified as *P. viridiflava* (Berge et al., 2014). These strains have been commonly found in environmental reservoirs with about 45% being Ice+ (Berge et al., 2014). We found 7 strains in phylogroup 9. All previous strains in phylogroup 9 have been found in aquatic habitats, but only 4% have been Ice+ (Berge et al., 2014). Berge et al. (2014) reported none of the samples in phylogroups 8, 11, 12, and 13 showed Ice+ activity. Since we sequenced the *cts* gene only for Ice+ strains, it is not surprising that none of the strains we collected were in these phylogroups, with the exception of phylogroup 13 to which four of our isolates belonged. We also examined the distribution of strains in different phylogroups according to sampling depth and found there was no correlation between sampling depth and phylogroups. All of the samples from the spring collections were in phylogroup 2, although the spring collections had so few Ice+ strains that it was difficult to examine their diversity.

A majority of the *Pseudomonas* Ice+ strains were *P. syringae*, which is consistent with previous studies indicating *P. syringae* is the most wide spread biological ice nucleator (Fall and Fall, 1998; Morris et al., 2011; Murray et al., 2012). Many of the non- *P. syringae* colonies showed the highest match with *P. fluorescens* when blasted against the NCBI database. *P. fluorescens* has previously been reported to have the Ice+ phenotype as well (Warren and Corotto, 1989).

This study indicates that freshwater lakes may be significant reservoirs of Ice+ *P. syringae*. Future work should examine the abiotic and biotic factors driving the potential selection of ice-nucleating strains of *P. syringae* in freshwater systems. Bacteria at or near the surface of lakes have the potential to aerosolize through crashing waves, wind sweeping across the surface of the water, and via rainsplash. Bacteria at greater depths within lakes also have the potential to move to the surface; strains of *P. syringae* contain flagella and may swim short distances and/or be moved through currents and seasonal turnover. Spring and fall turnover creates mixing throughout all the vertical profile of the lake that can bring new bacteria to the surface. Consequently, these bacteria have the potential to move to other parts of the water cycle and into the atmosphere (Morris et al., 2008), and future work should also focus on the processes involved in the aerosolization of these strains from aquatic environments.

## Author contributions

RP: designed and conducted experiments, analyzed data, and led the writing of the manuscript. BV: advised the experiment design and provided technical advice related to the sequencing, analyzed data, and assisted in writing the manuscript. DS: managed the project, designed experiments, assisted with sample collection, analyzed data, and assisted in writing the manuscript.

## Funding

This research was supported in part by the National Science Foundation (NSF) under Grant Numbers DEB-1241068 [Dimensions: Collaborative Research: Research on Airborne Ice-Nucleating Species (RAINS)] and DGE-0966125 [IGERT: MultiScale Transport in Environmental and Physiological Systems (MultiSTEPS)]. Any opinions, findings, and conclusions or recommendations expressed in this material are those of the authors and do not necessarily reflect the views of the National Science Foundation.

### Conflict of interest statement

The authors declare that the research was conducted in the absence of any commercial or financial relationships that could be construed as a potential conflict of interest.
